# Reduced neural connectivity in the caudate anterior head predicts hallucination severity in schizophrenia

**DOI:** 10.1016/j.schres.2023.08.030

**Published:** 2023-09-06

**Authors:** Leighton B.N. Hinkley, Shalaila S. Haas, Steven W. Cheung, Srikantan S. Nagarajan, Karuna Subramaniam

**Affiliations:** a Department of Radiology and Biomedical Imaging, University of California, San Francisco, CA 94143, USA; b Department of Psychiatry, Icahn School of Medicine at Mount Sinai, NY 10029, USA; c Department of Otolaryngology-Head and Neck Surgery, University of California, San Francisco, CA 94143, USA; d Surgical Services, San Francisco Veterans Health Care System, San Francisco, CA 94121, USA; e Department of Psychiatry and Behavioral Sciences, University of California, San Francisco, CA 94143, USA

**Keywords:** Schizophrenia, Hallucinations, Resting-state fMRI, Caudate

## Abstract

**Background:**

Caudate functional abnormalities have been identified as one critical neural substrate underlying sensory gating impairments that lead to auditory phantom hallucinations in both patients with schizophrenia (SZ) and tinnitus, characterized by the perception of internally generated sounds in the absence of external environmental auditory stimuli. In this study, we tested the hypothesis as to whether functional connectivity abnormalities in distinct caudate subdivisions implicated in sensory gating and auditory phantom percepts in tinnitus, which are currently being localized for neuromodulation targeting using deep brain stimulation techniques, would be associated with auditory phantom hallucination severity in SZ.

**Methods:**

Twenty five SZ and twenty eight demographically-matched healthy control (HC) participants, completed this fMRI resting-state study and clinical assessments.

**Results:**

Between-group seed-to-voxel analyses revealed only one region, the caudate anterior head, which showed reduced functional connectivity with the thalamus that survived whole-brain multiple comparison corrections. Importantly, connectivity between the caudate anterior head with thalamus negatively correlated with hallucination severity.

**Conclusions:**

In the present study, we deliver the first evidence of caudate subdivision specificity for the neural pathophysiology underlying hallucinations in schizophrenia within a sensory gating framework that has been developed for auditory phantoms in patients with tinnitus. Our findings provide transdiagnostic convergent evidence for the role of the caudate in the gating of auditory phantom hallucinations, observed across patients with SZ and tinnitus by specifying the anterior caudate division is key to mediation of hallucinations, and creating a path towards personalized treatment approaches to arrest auditory phantom hallucinations from reaching perceptual awareness.

## 1. Introduction

Patients with schizophrenia (SZ) suffer from profound positive psychotic symptoms, such as auditory verbal hallucinations (AVH), which often occur in the form of spoken words and are experienced in the absence of external auditory stimuli (Ford, 2016). SZ typically misattribute hallucinations as being externally-derived voices, and as being separate and distinct from their own inner self-generated thoughts, revealing severe impairments in self-agency (i.e., impairments in the awareness of being the originator of one’s own thoughts and actions) (Ford, 2016). One theory is that such impairments in self-agency that manifest as AVH symptoms may result from sensory gating impairments, described as an inability to filter out distracting and irrelevant information from internal and external sensory sources, and likely represents a central component of the underlying pathophysiology of hallucinations (Hazlett et al., 2008). One possible locus of this sensory gating impairment is the caudate nucleus, a region of the basal ganglia that plays a critical role in sensory gating (Hazlett et al., 2008). Typically, the caudate interfaces with multiple subdivisions of the basal ganglia (globus pallidus, subthalamic nucleus) and acts as a “gate” for receiving sensory inputs from the thalamus to select salient and relevant information and send to temporal, parietal and prefrontal cortices underlying working-memory (Chatham et al., 2014). Ample evidence exists not only for dysfunctional caudate structure and function in SZ, but also for its relation to hallucinations, suggesting that caudate functional abnormalities may underlie the pathophysiology of hallucinations in SZ (He et al., 2021; Perez-Costas et al., 2010; Duan et al., 2015; Koreki et al., 2019). Caudate functional abnormalities are also a distinguishing feature in patients with tinnitus, who experience bothersome auditory phantom percepts, posited to be the consequence of impaired sensory gating of internal auditory sound sources by the striatum (Hinkley et al., 2022; Cheung et al., 2019).

SZ and tinnitus patients both show reduced structural volumes in the caudate as well as disrupted structural and functional connections between the caudate and other brain regions (Koreki et al., 2019; Hinkley et al., 2022; Ebdrup et al., 2010; Venkatasubramanian et al., 2003). Convergent findings from neuroimaging and electrical stimulation studies indicate abnormal caudate functional connectivity plays a critical role in in inducing sensory gating impairments and triggering auditory phantom percepts in the absence of external environmental auditory stimuli (Hinkley et al., 2022; Cheung et al., 2019). Abnormal caudate connectivity is also considered a hallmark of auditory phantom percepts in tinnitus patients in which neuromodulation by deep brain surgery has been found to alter the perception of auditory phantoms (Hinkley et al., 2022; Cheung et al., 2019; Perez et al., 2019; Cheung and Larson, 2010). Taking these findings together, we hypothesized abnormal caudate connectivity may underlie disturbances in self-agency, resulting in symptoms of auditory phantom hallucinations in both patients in tinnitus and SZ (Koreki et al., 2019; Roberts et al., 2009).

The experience of self-agency is thought to be driven by the ability to make reliable predictions about the expected outcome of one’s own thoughts and actions (Subramaniam et al., 2018). A common example that Haggard (Haggard, 2017) illustrates to clearly demonstrate the importance of this concept is: If I turn on a light switch and the light comes on immediately, this results in the normal familiar experience of my being the agent of my actions of turning on the light. However, if the light comes on before I turn on the switch or if there is a delay between my turning on the switch and the light turning on, I experience a violation of my predictions (i.e., self-predictions) as a result of the mismatch between my predicted outcome and the actual action outcome, which results in my experience of self-agency being lost and the outcome is registered as externally-caused. Patients with schizophrenia show cardinal deficits in self-agency that contribute to distortions in reality monitoring (impairments in distinguishing self-generated from externally-produced events) (Subramaniam et al., 2012; Subramaniam et al., 2017; Subramaniam and Vinogradov, 2013; Subramaniam et al., 2016). For instance, symptoms of hallucinations are thought to arise from the misattribution of patients’ inner self-generated thoughts as externally-derived voices (Voss et al., 2010). These psychotic symptoms of hallucinations are thought to result from impaired self-predictions about the expected outcome of one’s own actions (Subramaniam et al., 2018; Voss et al., 2010; Synofzik et al., 2010; Synofzik et al., 2013; Subramaniam, 2021). Thus, the pathophysiology of hallucinations suggest SZ manifest weakened reliance on self-predictions about their own action outcomes, misattributing their own thoughts and actions as being externally-derived, resulting in their lost sense of self-agency.

The caudate is one region that is thought to be an integral component that mediates the self-prediction mechanisms underlying agency. The caudate is rich in dopaminergic innervations, that are necessary for modulating this self-prediction ability to enable individuals to make reliable predictions about the expected outcome of one’s own actions (Koreki et al., 2019; Roberts et al., 2009). In this sense, the caudate is thought to play a fundamental role in the emergence of self-agency, that results from a minimal prediction error between the predicted sensory consequence of a self-generated action and the actual outcome (Koreki et al., 2019). In SZ however, the caudate is considered to be one critical region, whose aberrant functional connectivity with other critical sensory gating regions, such as the thalamus, is thought to result in an impaired sense of agency, and the trigger of hallucinations (Koreki et al., 2019; Alitto and Usrey, 2003). For example, the dopamine hypothesis in SZ advocates that abnormal DA densities in the caudate observed in postmortem studies leads to impairments in information-gating, resulting in more permissive lax gating that may lead to an overflow of ‘noisier’ irrelevant information from internal sources during inner speech, making it more difficult for SZ to make reliable self-predictions about the expected outcome of one’s own actions, in order to generate self-agency judgments (Koreki et al., 2019; Howes et al., 2009; Howes and Kapur, 2009; Howes and Nour, 2016; Abi-Dargham et al., 1998; Kegeles et al., 2010; Gjedde and Wong, 1987).

One powerful way to understand whether a given anatomical region *causally* underlies a specific brain function or symptom (e.g. psychotic symptoms such as hallucinations) is to examine lesion studies. While psychotic symptoms following brain lesions are rare, prior case reports have provided evidence of patients without previous psychiatric disorders who developed psychotic symptoms following caudate infarction (Santos et al., 2009; Haas et al., 2021a). These studies revealed that the caudate abnormalities represent a critical neural correlate underlying the pathogenesis of hallucinations (Santos et al., 2009; Haas et al., 2021a). Together, these studies provide transdiagnostic convergent evidence across distinct lesion, postmortem, neuroimaging, molecular PET imaging and neuromodulation studies for the role of the caudate in the gating of auditory phantom hallucinations (Koreki et al., 2019; Hinkley et al., 2022; Cheung et al., 2019; Roberts et al., 2009; Howes et al., 2009; Howes and Kapur, 2009; Howes and Nour, 2016). In light of these prior convergent findings, here, for the first time, we tested the hypothesis as to whether functional connectivity abnormalities in distinct caudate subdivisions implicated in sensory gating and auditory phantom percepts in tinnitus which are currently being localized for neuromodulation targeting at the individual level using deep brain stimulation trials (Hinkley et al., 2022), would be associated with auditory phantom hallucination severity in SZ.

Here, we asked participants to completed resting-state scans with eyes-closed to understand the neural trigger of auditory phantom hallucinations in the absence of external sensory stimuli. In this way, we would be able to isolate neural aberrations underlying impairments in patients’ gating of irrelevant information from internal sources, that are thought to lead to misattribution of inner thoughts and inner ‘speech’ as external auditory phantoms, resulting in their impaired sense of agency and manifestation of hallucinations. Specifically, in the present study, we applied precision-medicine guided resting-state functional biomarkers in SZ within the specific caudate subdivisions that showed abnormal connectivity for identifying deep brain stimulation treatment targets from our current trials that are underway in patients with tinnitus (Cheung et al., 2019). We applied the methods of Jung et al. (Jung et al., 2014) to parcellate the striatum in SZ into functionally-defined subdivisions, in which we characterized striatal connectivity profiles using data-driven resting-state fMRI unsupervised clustering algorithms that were not constrained by pre-defined *apriori* anatomical or functional targets. We tested the hypothesis that functional connectivity abnormalities in these distinct caudate subdivisions implicated in auditory phantom percepts in tinnitus, would also be associated with auditory phantom hallucination severity in SZ.

## 2. Material and methods

### 2.1. Participants

This study represents the baseline fMRI resting-state portion of a NIMH-funded R01 (R01MH122897) study in schizophrenia. SZ participants were recruited from community mental health centers and outpatient clinics, and HC subjects were recruited via advertisement.

SZ (N = 25) and healthy control (HC) (N = 28) participants, matched at a group level on age and gender, gave informed consent for this IRB-approved protocol and completed this fMRI study and clinical assessments (see Table 1). Inclusion criteria were Axis I schizophrenia diagnosis, assessed with the DSM-V [SCID] or, for HC, no psychiatric disorder, no substance abuse, and age between 18 and 60 years. Hallucination severity was assayed using a subscale of the Positive and Negative Syndrome Scale (PANSS) on a scale of 1 (absent) to 7 (severe). Participants completed the PANSS immediately prior to the resting-state fMRI scan (see Table 1 for PANSS subscores).

### 2.2. MRI protocol

Participants underwent T1-weighted imaging and resting-state (eyes-closed) EPI in a 3 T Siemens scanner. Whole-brain structural MRI data were acquired using the following parameters (3Tesla, 3D sequence, flip angle 9°, TE 2.98 ms, TR 2300 ms, TI 900 ms, FOV 256 × 256 mm, matrix 256 × 256 × 252, NEX = 0.5, voxel dimensions 1 × 1 × 1 mm, slice thickness = 1, slices per tab = 160, acquisition time = 4:54 min). Resting-state scans were acquired with the following parameters (32 slices, slice thickness = 3.5 mm flip angle = 75°, TE = 29 ms, TR = 2000 ms, FOV = 240 × 240 mm).

### 2.3. Imaging analyses

Data were preprocessed using SPM12, and functional connectivity metrics were computed using CONN v20b (http://www.nitrc.org/projects/conn). Resting-state fMRI data were preprocessed using CONN (e.g. artifact detection (ART), motion correction and scrubbing, smoothing at 8 FWHM). Nine caudate subdivisions were identified using existing data-driven parcellations of functionally-defined subdivisions (Jung et al., 2014) and seeds (5 mm-radius spheres) were defined and generated for each subdivision using MarsBar to compute the magnitude of connectivity with all voxels in the brain, thresholded at p < .001, for each subject. All group functional connectivity analyses were performed using Fisher-Z transformed correlations to examine which caudate ROIs showed significant connectivity effects with all voxels in the brain, using multiple comparisons correction (FDR, p < .05). We used Spearman’s correlations to assay the strength of associations between ranked values of hallucination severity scores and connectivity in the caudate regions that showed significant between-group functional connectivity differences in SZ compared to HC.

## 3. Results

**Fig. 1.**
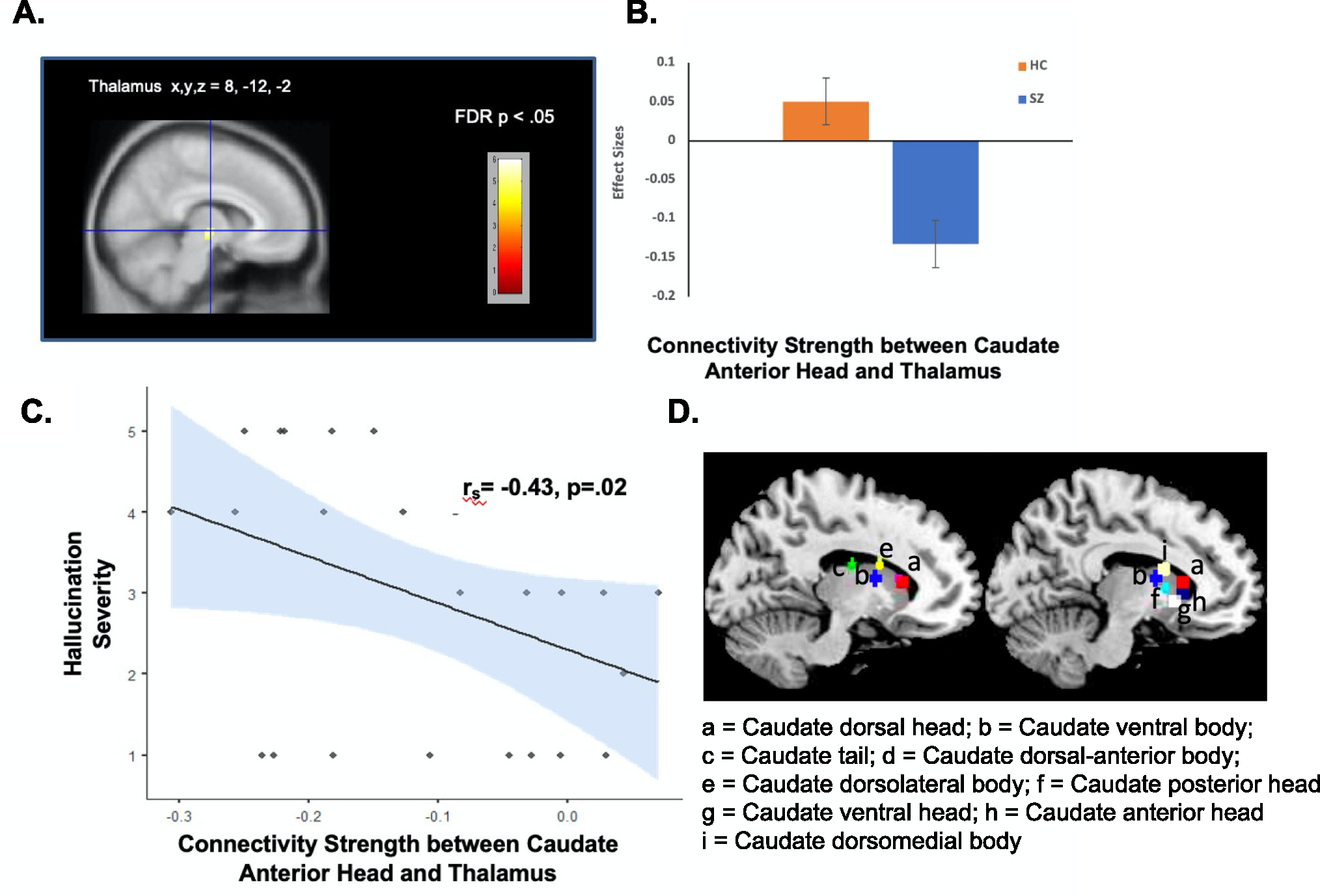
Between-group seed-to-voxel whole-brain analyses shows significantly reduced connectivity in SZ compared to HC, between only one seed region, the caudate anterior head seed with thalamus voxels (A & B; FDR, p < .05), that predicted worsening hallucination severity in SZ (C). D shows all nine caudate seed ROIs of which only the caudate anterior head showed significantly reduced whole-brain connectivity in SZ compared to HC with the thalamus.

Between-group seed-to-voxel analyses in SZ vs. HC, performed on the average z-maps between each caudate seed with all voxels in the brain revealed that only one region, the caudate anterior head, showed reduced functional connectivity with the thalamus that survived whole-brain multiple corrections (FDR p < .05; Fig. 1). Importantly, connectivity between the caudate anterior head with thalamus negatively correlated with hallucination severity (r_s_ = −0.43, p = .02; Fig. 1; Table 1).

Our trained clinical psychologists noted that while some patients also had visual and somatic hallucinations, SZ were all unified in manifesting auditory hallucinations. In our SZ sample, participants had hallucination severity ratings from 1 (absent) to 5 (moderately severe) (Table 1). When we removed the SZ subjects who had no hallucinations (i.e., scores of 1), we found a much stronger correlation between reduced caudate connectivity and hallucination severity (r_s_ = −0.78, p = .0001) (see Supplementary Fig. 1). We also found a correlation between chlorpromazine equivalents and hallucination severity (r_s_ = 0.46, p = .04). We did not find correlations between caudate connectivity in any seed with other symptoms (e.g. positive, negative or disorganized), medication or illness duration (all p’s > .30). Collectively, these findings indicate that aberrations in connectivity in the caudate anterior head and thalamus strongly predicted greater hallucination severity in SZ.

## 4. Discussion

We investigated the role of the caudate as a gating mechanism for auditory phantom hallucinations in SZ. This is the first study to reveal that weakened connectivity of a specific caudate subregion, the anterior caudate head, not only had significantly weaker connections with the thalamus in patients with SZ but that the weakened magnitude of these connections were significantly associated with hallucination severity. The present findings extend our prior work in tinnitus, where high-field resting-state fMRI revealed that specific caudate subdivisions, such as the caudate head, were associated with auditory phantoms, and are now being localized for precision-medicine based neuromodulation targeting at the individual level using deep brain stimulation (Hinkley et al., 2022). Collectively, these data provide pioneering transdiagnostic convergent evidence for a more generalized role of the caudate in the gating of auditory phantom percepts, observed across SZ and tinnitus.

Caudate functional abnormalities are a characteristic feature in both patients with SZ and tinnitus, in which patients experience auditory phantom hallucinations, characterized by the perception of internally generated sounds, in the absence of external auditory stimuli (Koreki et al., 2019; Hinkley et al., 2022; Cheung et al., 2019; Roberts et al., 2009). These findings motivated our utilization of resting-state neuroimaging techniques to understand the neural trigger of auditory phantom hallucinations in the absence of external sensory stimuli. The present findings extend prior work which have purported its functional disintegration with sensory gating regions, such as the caudate and thalamus, that underlie sensory gating impairments in filtering out irrelevant information from internal sensory sources, resulting in the misattribution of inner thoughts as external auditory phantom percepts (Koreki et al., 2019; Alitto and Usrey, 2003). The caudate is a critical pathological region which is thought to underlie agency impairments that result in auditory phantom hallucinations in SZ (Koreki et al., 2019; Abi-Dargham et al., 1998; Kegeles et al., 2010). In particular, DA dysfunction localized in the striatum has been proposed as one of the main neurotransmitter anomalies for contributing to gating impairments, resulting in psychotic symptoms (Koreki et al., 2019; Abi-Dargham et al., 1998; Kegeles et al., 2010). Specifically, DA dysfunction in the caudate permits “noisier” irrelevant information from internal neural sources during inner speech, which are routed via the thalamus to temporal, parietal and prefrontal cortices, making it much harder for SZ to generate accurate self-agency judgments, and more likely for SZ to misattribute internal ‘speech’ as external auditory phantom hallucinations (Koreki et al., 2019; Abi-Dargham et al., 1998; Kegeles et al., 2010; Gjedde and Wong, 1987).

The current findings move the field forward by providing the first evidence of localized connectivity deficits between the anterior caudate head and thalamus (e.g. ventral anterior and medial dorsal nuclei), with the weakened connectivity magnitude of this reduction being associated with more severe hallucinations. The thalamus is a fundamental way-station through which all sensory information is gated prior to reaching the prefrontal cortex (McCormick and Bal, 1994). The ventral anterior and medial dorsal nuclei of the thalamus, send and receive projections to sensory, motor and prefrontal regions, rendering the thalamus ideal for integrating sensory information to multiple sites throughout the brain (Pergola et al., 2015), but shows aberrant connectivity in SZ, where volumetric changes are also correlated with hallucinations (Portas et al., 1998). Importantly, prior reports also reveal that both infarctions to caudate and thalamus resulting from stroke to healthy participants, induced sudden-onset psychotic symptoms (Santos et al., 2009), suggesting that poor connectivity between the caudate and thalamus likely represents a neural biomarker for the pathogenesis of hallucinations. The results of the present study dovetail these previous findings in stroke and psychosis by suggesting that impoverished caudate-thalamic connectivity leads to auditory phantom percepts in schizophrenia as it does in these other disorders. Collectively, these datasets highlight the importance of impoverished sensory gating deficits between subcortical structures in psychosis-spectrum disorders.

There are some limitations of the present study. First, hallucination severity was only assayed with the PANSS immediately prior to the resting-state acquisition, and not with the addition of other clinical assessments. We note that while the PANSS is highly-validated and we expect there would be good temporal concordance in this time-frame (i.e., no change in hallucinatory reports) during the 5 minute resting scan, we did not interview patients on their hallucination occurrences during their resting state scan and thus we were not able to test their a posteriori hallucinatory reports after the resting-state scan (Leroy et al., 2017). Second, based on our a priori hypothesis, we expected and found specific caudate subdivisions that revealed abnormal connectivity that predicted more severe clinical psychopathology in SZ. It is certainly possible that abnormal connectivity in other seeds (e.g. nucleus accumbens) with other brain regions would also underlie hallucination percepts (Rolland et al., 2015). We conclude that poor connectivity in anterior caudate head represents one region (but not the only region), that underlies hallucination percepts in SZ. Third, we found a positive correlation between chlorpromazine (CPZ) equivalents and hallucination severity. Within this single time-point of merging clinical and neuroimaging data, the positive correlation between CPZ equivalents and hallucination severity in our chronically-ill SZ sample suggest that higher antipsychotic dosage was administered to treat greater hallucination severity, and that findings may be limited to our chronically-ill SZ sample who may have presented with more severe treatment-resistant hallucinations.

In summary, we provide a novel perspective for investigating the neural pathophysiology underlying hallucinations in SZ within a striatal gating framework that has been developed for auditory phantoms in tinnitus. In this first-of-its-kind report, we now deliver robust evidence revealing the caudate anterior head division is one region that contributes to the neural pathophysiology underlying hallucinations in schizophrenia. We have previously shown that it is possible to predict treatment response at the individual level using structural MRI and resting-state fMRI (Hinkley et al., 2022; Kambeitz-Ilankovic et al., 2021; Haas et al., 2021b). The next step in a larger sample would be to investigate the predictive accuracy of treatment response to deep brain stimulation for improving hallucination severity at the individual level using machine learning approaches based on resting state connectivity metrics and clinical measures, such as the PANSS, Psychotic Symptoms Rating Scales (PSYRATS) (Haddock et al., 1999) and Auditory Vocal Hallucination Rating Scale (AVHRS) (Bartels-Velthuis et al., 2012). In conclusion, our findings contribute to a larger body of literature on thalamic-striatal sensory gating dysfunction in schizophrenia by specifying the anterior caudate division is key to the mediation of hallucinations, and for creating a path towards personalized approaches to arrest auditory phantom hallucinations from reaching perceptual awareness.

## Supplementary Material

Supplementary Figure 1

## Figures and Tables

**Table 1:** Demographics and clinical profile (mean, SD) in healthy control participants (HC) and schizophrenia patients (SZ).

	SZ	HC

Age	45 (10.6)	43 (8.5)
Gender	20M, 5F	22M, 6F
Hallucination Severity	2.78 (1.6)	N/A
Positive Symptoms	2.56 (0.96)	N/A
Negative Symptoms	2.23 (0.94)	N/A
Disorganized Symptoms	1.99 (0.71)	N/A
Chlorpromazine (CPZ) Equivalents	321.8 (188.6)	N/A
Illness Duration (Years)	26 (11)	N/A
