# Supplementary material for: Reduced neural connectivity in the caudate anterior head predicts hallucination severity in schizophrenia
Source: Schizophr Res. Author manuscript; Available in PMC 2024 Feb 20. (PMC10878029; doi:10.1016/j.schres.2023.08.030)
Supplement: Supplementary Figure 1 [file NIHMS1963159-supplement-Supplementary_Figure_1.pdf]

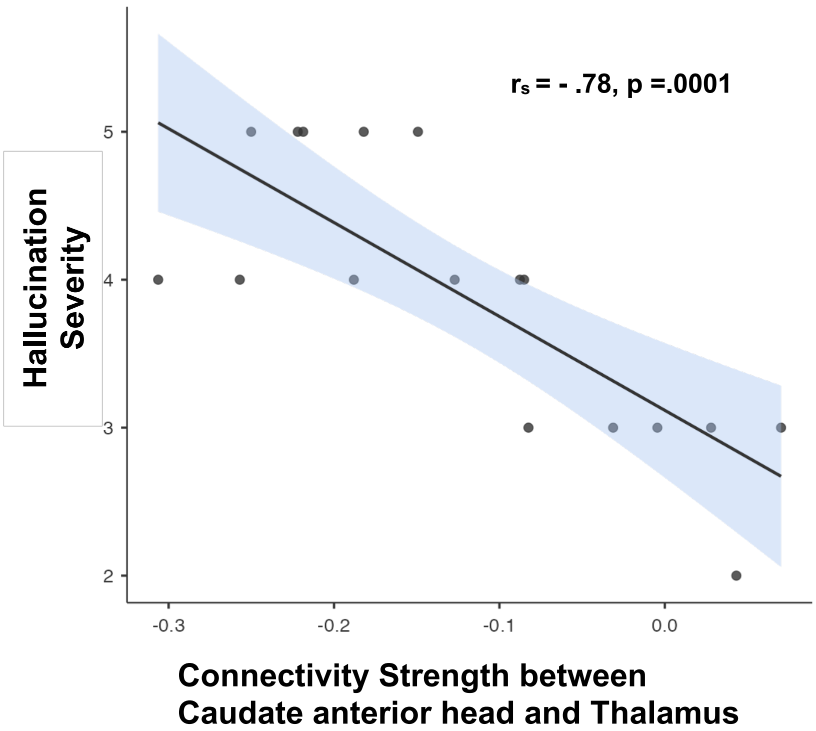

Supplementary Figure 1. Between-group seed-to-voxel whole-brain analyses shows significantly reduced connectivity in SZ compared to HC, between the caudate anterior head seed with thalamus (FDR, $p<.05$), that strongly predicted worsening hallucination severity, after removing participants with scores of 1 who did not manifest hallucinations.
